# Advances in the Molecular Mechanisms of Abscisic Acid and Gibberellins Functions in Plants 2.0

**DOI:** 10.3390/ijms23158524

**Published:** 2022-07-31

**Authors:** Víctor Quesada

**Affiliations:** Instituto de Bioingeniería, Universidad Miguel Hernández, Campus de Elche, 03202 Elche, Spain; vquesada@umh.es; Tel.: +34-96-665-8812

Abscisic acid (ABA) and gibberellins (GA) are two important hormones that antagonistically regulate many aspects of plant growth and development. These antagonistic regulatory functions of ABA and GA involve different plant development stages, i.e., seed maturation and dormancy, hypocotyl elongation, root growth and flowering [1]. In a broad sense, GA and ABA respectively promote and inhibit cell elongation and growth. Noteworthily, several factors mediate ABA and GA antagonism; for instance, regulating the transcription patterns of ABA and GA biosynthesis genes and, hence, the balance between ABA and GA [2]. Apart from playing a key role in regulating several physiological and developmental processes, ABA has also been traditionally considered the plant stress hormone par excellence. Accordingly, ABA directly responds to different adverse environmental conditions (i.e., low temperatures, drought, salinity or flooding) through the ABA signal transduction pathway, which results in massive changes in gene expression [3], stomatal closure [4] and lower transpiration rates [5,6]. Interestingly, recent data suggest that GA are also involved in plant response to environmental stress [7,8].

This Special Issue is the continuation of the previous Special Issue “Advances in the Molecular Mechanisms of Abscisic Acid and Gibberellins Functions in Plants”. It contains six original research articles and four reviews published by field experts. These works cover outstanding advances in the molecular mechanisms by which GA and ABA control different aspects of plant physiology, development and for ABA, also in the response to abiotic stresses. In line with this, the original research articles investigate the effects of ABA on epicuticular wax metabolism regulation in a citrus fruit cultivar [9], the regulation of WNK kinases and their comprehensive interaction with ABA components [10], CCD family members in poplar and their expression patterns in response to ABA and abiotic stress [11], the molecular function of NAC transcription factor PwNAC11 in drought stress [12], the interaction between AFPs and DELLA proteins [13], and the molecular mechanism by which AGB1 regulates the GA pathway in Arabidopsis [14]. The reviews published in this Special Issue focus on how ABA regulates plant development and stress tolerance through alternative splicing [15], the mechanisms of ABA-mediated drought stress responses in plants [16], the roles of ABA and GA in stem/root tuber development [17] and the molecular mechanisms underlying ABA and GA regulation of seed development from embryogenesis to maturation [18]. In this editorial, I sum up the most significant findings of these insightful works.

The research article by Romero and Lafuente [9] exhaustively investigated the effects of ABA on epicuticular wax metabolism regulation in a mutant citrus cultivar named Pinalate (*Citrus sinensis* L. Osbeck), defective in ABA-biosynthesis and, hence, with low ABA levels. To this end, Pinalate sweet orange fruits were harvested, treated with ABA and then exposed to water stress by storing them at low relative humidity. Romero and Lafuente [9] reported that ABA treatment modifies the composition and metabolism of the epicuticular wax of Pinalate fruit after detachment, which depends on whether fruit is exposed, or not, to postharvest dehydration, and also on water stress duration. Remarkably, the epicuticular wax load in Pinalate fruit increases after detachment, whereas ABA exposure is able to attenuate this increase. Finally, the study of the expression profile of the key genes involved in wax metabolism revealed that they are all influenced by ABA to a certain extent. Romero and Lafuente [9] highlighted that the obtained results can improve current knowledge about ABA regulation of cuticular wax metabolism in fruit and might be useful for industrial wax synthesis.

WNK [With-No-Lysine (K)] kinases are serine-threonine kinases. They are so named because the K residue present in subdomain II of most kinases is not conserved in WNK kinases. Instead, the K residue is replaced with a cysteine residue [19]. WNK kinases have been found in several eukaryotes, including plants where the WNK gene family is larger and more diverse than in animals. Plant WNK kinases are involved in not only the regulation of various physiological and developmental processes, but also in the response to different abiotic and biotic stresses. In line with this, some WNK family members have been implicated in the ABA-signaling pathway. Accordingly, *Arabidopsis thaliana* (hereafter Arabidopsis) WNK8 kinase functions as a negative regulator of ABA signaling. Interestingly, the presence of a conserved peptide encoded by an upstream open reading frame (uORF) called CPuORF58 has been reported in the 5′-UTR of *WNK8* [20]. Li et al. [10] thoroughly studied *WNK8* regulation and interaction with ABA components. They indicated that WNK8 negatively regulates ABA response during seed germination and post-germination development. Additionally, the use of several genetic constructs bearing different mutations in the 5′-UTR of *WNK8* revealed that CPuORF58 is essential for the translational suppression of WNK8, likely because this uORF causes ribosome stalling. Another analysis showed that *WNK8* 5′-UTR actually contains two short open reading frames, CPuORF58 and uORF1, and both are required for *WNK8* expression. When mutant *wnk8-1* plants are transformed with the native *WNK8* promoter that drives *WNK8* expression, they exhibit a similar phenotype to that of the wild type, whereas the plants transformed with a similar construct, but mutated in CPuORF58 translation initiation codon ATG, display much less sensitivity to ABA. Furthermore, WNK8 and its downstream target RACK1 (receptor for activated C kinase1) synergistically coordinate ABA signaling. Li et al. [10] concluded that *WNK8* post-transcriptional regulation by the CPuORF58 conserved peptide located in its 5′-UTR is required for accomplishing plant ABA requirements. 

Carotenoid cleavage dioxygenases (CCDs) catalyze the cleavage of several carotenoids in higher plants, which results in the biosynthesis of biologically smaller apocarotenoids, including phytohormones such as ABA and signaling molecules such as β-cyclocitral, which play important roles in regulating plant growth, development and stress response [21]. Despite the significance of plant CCDs, the CCD gene family has not been hitherto studied in poplar (*Populus trichocarpa*), a species used as a model system in plant research with a significant ecological value. Wei et al. [11] reported an extensive genome-wide analysis of the CCD family of genes in poplar (*PtCCDs*). These authors systematically studied: *PtCCD* gene structures and conserved motifs; the expansion and contraction of *PtCCD* genes; the presence of *cis*-acting elements; the three-dimensional structures of PtCCD proteins and interaction networks. Wei et al. [11] evaluated the transcript profiles of *PtCCDs* in several tissues and determined the expression profile of *PtCCDs* in response to ABA and abiotic stress. They noted that *PtCCD* genes exhibit diverse tissue-specific expression patterns and, in response to abiotic stress and ABA, different *PtCCD* members display divergent response profiles. These results suggest that *PtCCD* genes may be involved in numerous physiological processes and in tolerance to both ABA and abiotic stress.

The NAC (NAM, ATAF1/2, and CUC2) transcription factors (TFs) in different plant species play important roles in the response to adverse environmental conditions. Notwithstanding, knowledge about NAC TFs involvement in the response of coniferous forests to abiotic stress is still scarce. To advance in this field, Yu et al. [12] performed a comprehensive functional analysis of the *NAC11* gene of the coniferous species *Picea wilsonii* in relation to drought tolerance. They reported that *PwNAC11* is significantly up-regulated by drought at 3 h [treatment with polyethylene glycol (PEG)] or ABA exposure in *P. wilsonii* seedlings, which suggests that PwNAC11 probably participates in early responses to stress. Later, Yu et al. [12] obtained *PwNAC11* overexpression (OE) lines in Arabidopsis and carried out some experimental assays to investigate the PwNAC11 function in drought stress. Along these lines, under drought conditions the Arabidopsis *PwNAC11* OE lines show enhanced drought tolerance and increased photoprotection and reactive oxygen species (ROS) scavenging ability. Moreover, *PwNAC11* OE enhances the expression of stress- and ABA-responsive genes under drought conditions. In line with the up-regulation of *PwNAC11* with exogenous ABA treatment in *P. wilsonii*, Yu et al. [12] found that *PwNAC11* OE increases ABA sensitivity and promotes ABA-induced stomatal closure in Arabidopsis. These findings indicate that *PwNAC11* OE lines are more sensitive to exogenous ABA. Furthermore, the authors searched for PwNAC11 potential interactors by performing yeast two-hybrid and bimolecular fluorescence complementation assay to find that PwNAC11 interacts in vivo with ABA-induced protein ABRE Binding Factor3 (ABF3) and ABA-independent DRE-BINDING PROTEIN 2A (DREB2A). Furthermore, the results obtained by Yu et al. [12] showed that PwNAC11 cooperates with ABF3 and DREB2 to activate *ERD1* transcription through the binding to the ABRE and DRE motifs of the *ERD1* promoter, respectively. These authors propose that PwNAC11 might mediate drought stress response via ABA-dependent and ABA-independent pathways.

The antagonistic effects of ABA and GA, respectively repressing and promoting seed dormancy and germination, are mediated by a complex network of positive and negative regulators of transcription. Two sets of negative regulators are the DELLA proteins and the AFP family of ABI5 [ABA-insensitive (ABI)]-binding proteins. DELLAs and AFPs are repressors of the GA and ABA response, respectively. Extensive interactions between ABI5, on the one hand, and AFPs and DELLAs on the other hand, have been reported. In their work, Finkelstein and Lynch [13] explored the possibility of an interaction between DELLAs and AFP proteins. By using yeast two-hybrid and bimolecular fluorescence complementation assays, direct interactions at different levels of intensity between DELLA and AFP family members were detected. Finkelstein and Lynch [13] also investigated potential genetic interactions between AFPs and DELLA proteins. They found that the overexpression (OE) of AFP proteins in a *sleepy1* (*sly1*) mutant background, a hyperdormant mutant where the GA response is repressed due to the overaccumulation of DELLA proteins, suppresses *sly1* hyperdormancy and hypersensitivity to ABA. However, AFP OE does not modify the dwarf and poor fertility phenotype of the *sly* mutant, but brings about a reduction in the accumulation of the seed storage proteins associated with the *sly* mutation. Finkelstein and Lynch [13] propose that the reported interactions are suggestive of additive effects of AFPs and DELLAs, which is consistent with these proteins acting in convergent pathways.

Unlike the above-discussed research manuscripts, which deal with different aspects of ABA, or the ABA and GA function, in plants, the work of Qi et al. [14] focused on GA, and specifically on two components of the GA pathway: Arabidopsis G-protein β subunit (AGB1) and the DNA-binding protein MYB62, a GA pathway suppressor. Heterotrimeric G proteins are transmembrane proteins that transduce signals from a wide range of extracellular stimuli and regulate several cellular and physiological functions in eukaryotes. In plants, G proteins are involved in developmental processes, stress responses and innate immunity [22]. Qi et al. [14] identified mutants *agb1-2* and N692967 that are affected in AGB1, which are dwarfs and contain significantly reduced GA levels compared to the wild type when undergoing GA_3_ treatment. By using yeast-two hybrid, pull-down and firefly luciferase complementary imaging, Qi et al. [14] found that AGB1 physically interacts with the DNA-binding region of GA pathway suppressor MYB62. The results of the genetic analysis carried out by Qi et al. [14] are consistent with MYB62 acting downstream of AGB1 in the GA pathway. The expression analysis performed by RT-qPCR and competitive DNA binding assays revealed that MYB62 can bind MYB elements in the promoter of downstream gene *GA2ox7* (encoding a GA degradation enzyme) to induce *GA2ox7* transcription. Interestingly, the interaction of AGB1 with the DNA-binding region of MYB62 inhibits the binding of MYB62 on the *GA2ox7* promoter. These results reveal that AGB1 functions as a negative regulator of MYB62 activity and, consequently, positively regulates the GA pathway in Arabidopsis.

In eukaryotic cells, alternative splicing (AS) generates different mature mRNAs from the same mRNA precursor by selecting distinct combinations of splicing sites. In this way, AS increases the diversity and complexity of eukaryotic transcriptomes and proteomes. In plants, AS is extensively involved in not only the hasty regulation of plant growth and development, but also in adaptation to adverse environmental conditions. However, the mechanisms by which ABA can regulate plant development and tolerance to abiotic stress by mediating AS are not well understood. Yang et al. [15] reported in their review that ABA mediates plant abiotic stress tolerance and development, particularly through AS events. First, the authors briefly revised the main roles of ABA in abiotic stress response, the regulatory network of ABA signaling and ABA functions in development by focusing on seed germination and flowering. Next, they showed that ABA-induced AS occurs primarily in regulatory genes, such as transcription factors, protein kinases and splicing factors, as well as in genes of the ABA signaling pathway in Arabidopsis. Yang et al. [15] highlighted that ABA affects the splicing of genes of its own signaling pathway and other genes, especially by regulating the expression of splicing factors, such as SR genes and U2AF. They reported that this results in different splicing isoforms by, thereby, increasing protein abundance and improving plant adaptation to harsh environmental conditions.

Drought severely impairs plant growth and agriculture production, which are especially relevant under current climate change conditions. ABA is a key hormone that controls plant responses to drought through complex molecular signaling mechanisms. In line with this, ABA is able to synchronize a wide range of functions in plants by helping to overcome drought stress. Muhammad Aslam et al. [16] exhaustively worked on summarizing current knowledge about ABA-mediated drought responses through physiological, biochemical and root system alterations. In their review, Muhammad Aslam et al. [16] also highlighted the molecular mechanism of ABA-mediated drought regulation (i.e., ABA-dependent translational and post-translational modifications) as well as ABA crosstalk with other hormones, including auxins, GA, cytokinins, ethylene, salicylic acid and jasmonic acid, required to help plants to cope with drought and other abiotic stresses.

The remaining two reviews included in this Special Issue have focused on the roles of ABA and GA in stem/root tuber development [17] and seed germination [18] by highlighting molecular aspects. In the first case, the paper by Chen et al. [17] provides a comprehensive overview of recent advances in understanding ABA and GA functions in the stem/root tuber development of different tuber crops. Root and tuber crops, such as potato (*Solanum tuberosum*), sweet potato (*Ipomoea batatas*) and cassava (*Manihot esculenta*), are important for human food given their underground storage organs that are rich in water, carbohydrates and a range of proteins and secondary metabolites. Chen et al. [17] revised the roles of ABA and GA metabolism and signaling pathways in stem/root tuber development by showing that these phytohormones perform antagonistic functions in stem/root tuber development. In this way, ABA stimulates tuber formation, whereas GA represses tuber swelling. Therefore, a higher GA/ABA ratio results in longer stems and delayed tuberization, while a higher ABA/GA ratio leads to tuberization.

Seed development can be divided into two main phases, embryogenesis and maturation, which end with seeds entering dormancy to leave them ready to germinate when environmental conditions are favorable and after dormancy breaks. Complex gene regulatory networks govern seed development and germination, which involve several phytohormones. Of them, ABA and GA are the main hormones that antagonistically regulate seed development and germination. Although much progress has been made in understanding the molecular mechanism of ABA and GA regulation of seed maturation, much less is known about the role of both hormones in embryogenesis. Kozaki and Aoyanagi [18] thoroughly summarized current knowledge about the intricate molecular gene networks that regulate gene seed development from embryogenesis to maturation (including the accumulation of seed storage products, desiccation tolerance and induction and maintenance of primary seed dormancy) by focusing especially on the function of GA and ABA in all these processes. Kozaki and Aoyanagi [18] emphasized the intricate crosstalk among signaling phytohormones, besides ABA and GA, during seed formation, which deserves to be further investigated to provide a more complete picture of the mechanism governing seed development.

I am convinced that the high quality of the original research articles and reviews published in this Special Issue will undoubtedly contribute to improve our understanding of the molecular mechanisms of ABA and GA functions in plants. I wish to thank all the authors for their contributions and the reviewers for their critical assessments of these articles. I also thank Assistant Editor Ms. Reyna Li for offering me the opportunity to serve “Advances in the Molecular Mechanisms of Abscisic Acid and Gibberellins Functions in Plants 2.0” as a Guest Editor.
